# The heterogeneous association between education and the adoption of safe food handling practices in Ethiopia

**DOI:** 10.1186/s13561-025-00601-2

**Published:** 2025-02-22

**Authors:** Kai Su, Barbara Kowalcyk, Devin LaPolt, Lina Gazu, Silvia Alonso, Binyam Moges Azmeraye, Desalegne Degefaw, Galana Mamo, Dessie Abebaw Angaw, Amete Mihret Teshale, Robert Scharff

**Affiliations:** 1 Department of Human Sciences, The Ohio State University, Columbus, OH USA; 2 Department of Food Science and Technology, The Ohio State University, Columbus, OH USA; 3 Translational Data Analytics Institute, The Ohio State University, Columbus, OH USA; 4 Milken Institute School of Public Health, George Washington University, Washington, DC USA; 5 Health Program, International Livestock Research Institute, Addis Ababa, Ethiopia; 6 The Ohio State University Global One Health Initiative, Eastern Africa Regional Office, Addis Ababa, Ethiopia; 7 School of Public Health, College of Health and Medical Science, Haramaya University, Harar, Ethiopia; 8 Department of Epidemiology and Biostatistics, College of Medicine and Health Sciences, Institute of Public Health, University of Gondar, Gondar, Ethiopia; 9 National Clinical Bacteriology and Mycology Reference Laboratory, Ethiopian Public Health Institute, Addis Ababa, Ethiopia

**Keywords:** Behavior bias, Education, Ethiopia, Food handling practice, Food safety, Grossman model, Low- and middle-income countries, Optimism bias, Protective health behavior, Risk

## Abstract

**Background:**

Foodborne disease is a great concern to low- and middle-income countries. To prevent illness and death, intervention strategies need to be implemented across the food safety system and should include promoting the adoption of safe food handling practices. The positive association between education and health has been well-established, and one possible mechanism is that education may improve health by encouraging individuals to adopt more appropriate protective practices. Decisions regarding adoption of these practices may also be influenced by the food safety risks individuals face, the trade-offs they make to maximize utility, or behavior biases which may be correlated with education. This study aims to estimate the heterogeneous association between education and the adoption of safe food handling practices among people facing different levels of food safety risk.

**Methods:**

Models were constructed based on the Grossman health model and risk as well as behavior bias theories. Multivariate logistic regression models were estimated to explore the heterogeneous associations using data from a community survey conducted in Ethiopia. Agricultural household status and livestock presence were used as proxies to represent varying risk levels. Average marginal effects were estimated to provide a more accessible interpretation of the results.

**Results:**

Results showed that the association between education and certain safe food handling practices was positive among individuals in households assumed to face higher food safety risks, while the association was less pronounced (or even negative) for those facing lower levels of risk. We observed that secondary education attainment was associated with a 20 percentage points increase (*p* < 0.01) in the probability of washing hands compared to the reference group (illiterate) in agricultural households. However, for non-agricultural households, secondary education was associated with a 10 percentage points decrease (*p* < 0.05) in probability. Similar patterns were found for washing surface.

**Conclusions:**

Education is associated with increased adoption of safe food handling practices among individuals facing higher food safety risks. This has important implications for developing targeted policies focused on individuals most susceptible to foodborne diseases. Future policies aimed at increasing the adoption of safe food handling practices should also integrate individuals' decision-making processes and behavior biases in the context of varying risk levels.

## Background

### Introduction

Foodborne diseases (FBD) are prevalent in low- and middle-income countries (LMICs), resulting in adverse economic consequences. It is estimated by the World Bank [1] that US$110 billion is lost each year in productivity and medical expenses resulting from unsafe food in LMICs. Ethiopia, listed as one of the least developed countries in the world by the United Nations [2], is facing significant food safety challenges due to insufficient infrastructure and regulatory deficiencies [3]. Ethiopia also has high levels of food insecurity [4], which can modify exposure and increase risk of negative foodborne illness outcomes [5].

The U.S. Centers for Disease Control and Prevention (CDC) suggests that the implementation of safe food handling practices can reduce food safety risks through cooking, avoiding cross-contamination, and preventing bacterial growth [6]. Inappropriate and inadequate food handling practices can increase the risk of FBD [7]. Therefore, it is imperative to understand the factors associated with better food handling practices. This study aims to estimate the association between education and food handling practices, and to determine whether there is heterogeneity in the association between education and food handling practices among people facing different levels of FBD risk using data from a cross-sectional community survey conducted in Ethiopia.

The role of education on health behaviors has been examined in both economics and psychology literatures. Economic studies show that there is a positive association between education and health. One possible mechanism is that knowledge learned in school allows students to understand and better implement the practices that should be adopted to improve health [8, 9]. Similarly, people with higher levels of education can seek out and use knowledge more efficiently [8–10]. This suggests that there is likely to be heterogeneity in the association between education and health behaviors, including safe food handling practices. This association is expected to be more pronounced for those living in higher FBD risk environments, such as individuals whose primary source of income is agriculture. These individuals are often exposed to foodborne pathogens via closer contact with animal waste, contaminated water, and unpasteurized products.

Optimism bias, which is defined as the difference between individuals' optimistic expectations and the actual outcomes of events [11], can also play a role in shaping the association between education and safe food handling knowledge, attitudes and practices. For example, optimism bias can lead people to believe that they are less likely to be sickened with FBD compared to others. A more detailed explanation is provided in the following section. Evans et al. [12] demonstrated a positive correlation between education and optimism bias. Consequently, education may be negatively associated with safe food handling practices, as individuals with higher levels of education might be overly optimistic about the actual outcomes of events. Since there are two forces that may affect the association between education and safe food handling practices in different ways (with unknown directions and magnitudes), it is important to empirically estimate these associations to understand the mechanisms driving the relationship between these two variables.

In this study, we estimated the association between education and four safe food handling practices that have been found to reduce the risk of FBD from animal-sourced foods: 1) handwashing; 2) washing surfaces; 3) boiling or filtering water used to prepare food; and 4) using separate utensils for vegetables and meat. To determine if the association varies between households with different levels of risk for contracting diarrheal diseases, we used agricultural households as our primary proxy for risk. This is supported by literature that demonstrates the presence of microbial contamination on farmworkers’ hands, which could then lead to cross-contamination [13, 14]. As a robustness check, we also examined livestock presence in the household as proxy for risk. This is supported by Klous et.al. [15], who reviewed the literature documenting the transmission of zoonotic pathogens due to human-livestock contacts.

### Review of theory and empirical studies

#### The association between education and health

The positive relationship between education and health is well established. Ross and Wu [16] found that the positive association between education and health is significant after controlling for other socio-economic explanatory variables. Lleras-Muney [17] found a causal effect of education on health by estimating the effect of education on adult mortality. Several hypotheses were developed based on Grossman’s Efficient Producer Theory that could explain the effect.

Grossman [8] proposed that individuals with higher levels of education are efficient producers of health. Therefore, people with more education should have more comprehensive knowledge on health, which could help promote their physical well-being [9, 10]. Meara [9] also hypothesized that, at a given level of knowledge, individuals with higher levels of education can use knowledge more efficiently and are more likely to respond to it than less-educated individuals.

Previous studies have empirically estimated these mechanisms. Hoffmann and Lutz [18] found that the Allocative Efficiency Theory could explain the positive relationship between education and health. Their results showed that the positive association between education and healthier lifestyles can be mostly explained by the knowledge gained through education attainment. Meara’s study [9], which estimated the effect of education on smoking during pregnancy, showed that decreases in the prevalence of smoking between births varied by education level. This indicates that education is associated with different responsiveness to knowledge that affects health behaviors.

A number of studies have examined the association between education and protective health behaviors, but the results are mixed. Rattay et al. [19] found educational differences in handwashing and social distancing compliance during the COVID-19 pandemic among people in Germany. They found that less-educated individuals were less likely to adhere to these practices. Studies in Ethiopia [20, 21] also showed an association between low education level and adherence to COVID protective practices. Yet, no difference has been found in some other studies [22, 23]. For example, Plohl and Musil [22] suggested that the lack of a significant correlation may be due to the limited variability in the education levels within their sample. Conversely, Duggan et al. [24] found a negative association between professional education and handwashing compliance among medical professionals.

A few studies have examined the relationship between education and safe food handling practices in Ethiopia. Some found a positive association between education and the probability of adopting safe food handling practices [25, 26]. These studies, however, were conducted in limited geographical areas (e.g., a single town or university), and did not focus on safe food handling practices at home. Thus, they may not be representative of household food handling practices in Ethiopia.

Although most studies focused on health behaviors have included socio-demographic variables (e.g., age, gender, marital status, and income) and household characteristics (e.g., source of water), few studies have examined the possible heterogeneity of educational effects. For example, Brand and Davis [27] found heterogeneous educational effects on fertility among women with different social backgrounds. The results showed that the negative association between education and the number of children born (i.e., individuals with higher levels education have fewer children) is less significant among those with more advantageous socio-economic backgrounds compared to those with disadvantaged backgrounds. Another study aimed at estimating gender differences in the relationship between education and adult mortality concluded that gender-based heterogeneity was not found in this relationship, suggesting that men do not consistently gain more from education than women [28]. Although the heterogeneity in the association between education and the adoption of safe food handling practices has not been examined among individuals with varying risk perceptions, studies conducted in other contexts suggest that it is meaningful to test the hypothesis that education may have different impacts across different groups.

#### The role of risk in determining the educational effect

Theoretically, the level of risk reduction associated with a mitigating action influences optimal health behavior. This study uses the following model to illustrate the dynamics of this process. First, each individual *i* faces probability *p* that they become sick due to FBD. This probability is affected by the extent to which the individual engages in healthy behaviors *B*_*i*_ (e.g., handwashing). Individual *i* experiences utility ${U}_{i}\left(I,{B}_{i}\right)$ if they become ill and ${V}_{i}\left(H,{B}_{i}\right)$ if they remain healthy. Equation (1) illustrates the resulting optimization problem.

1 $${Max}_Hp_{i\;}\left(B_i\right){\times U}_i\left(I,B_i\right)+(1-p_i\left(B_i\right)){\times V}_i\left(H,B_i\right)$$

Taking first order conditions and rearranging terms yields:

$$\frac{\partial {V}_{i}}{\partial {B}_{i}}-{p}_{i}\left(\frac{\partial {V}_{i}}{\partial {B}_{i}}-\frac{\partial {U}_{i}}{\partial {B}_{i}}\right)=\frac{\partial {p}_{i}}{\partial {B}_{i}}\times \left({V}_{i}-{U}_{i}\right)$$

Next, it is assumed that the only direct effect of behavior on utility is a cost incurred prior to the manifestation of risk ${p}_{i}$. Thus, $\partial {V}_{i}/\partial {B}_{i} \text{is expected to be equal to} \partial {U}_{i}/\partial {B}_{i}$*,* yielding the optimization rule in Eq. (2).

2 $$\frac{\partial {V}_{i}/\partial {B}_{i}}{\partial {p}_{i}/\partial {B}_{i}}={V}_{i}-{U}_{i}$$

This says that an individual will engage in safe behaviors until the ratio of marginal cost $(\partial {V}_{i}/\partial {B}_{i})$ to the marginal risk reduction $(\partial {p}_{i}/\partial {B}_{i})$ from risk mitigation behavior equals the difference in utility between the two health states. In the Grossman model, education is seen as helping individuals understand the marginal costs and benefits associated with adopting protective health behaviors, improving the efficiency of health decisions. While this is seen as improving overall health, the direction of effects for individual health behaviors is indeterminant, as multiple routes to better health are often available. More generally, the optimal amount of safe food handling practices adopted is not equivalent to maximum adoption due to resource limitations (see below).

Another characteristic of this model is that risk levels are not fixed. For example, the initial risk level for individual *i* may be lower than that for individual *j* (*p*_*i*_ < *p*_*j*_) if individual *i* lives in an environment with fewer vectors of contamination than individual *j* (e.g., if *j* is a farming household). For example, if proper handwashing leads to roughly equal final risk levels, the marginal effect of the behavior will be larger for those with higher initial levels of risk $(\partial {p}_{j}/\partial {B}_{j}>\partial {p}_{i}/\partial {B}_{i})$.

We assumed that agricultural households face a higher risk of FBD due to more frequent exposures to contaminated irrigation water, manure, and animal contact, as suggested in previous studies. Ali et al. [29] found that wearing farm clothing and bringing unwashed farm tools into home were risk factors associated with diarrhea, a major symptom of FBD, among those using contaminated irrigation water. This study indicated that pathogens from irrigation water can be transmitted from the outside environment into home and affect other household members if proper sanitary practices are not followed. Contaminants brought in from the farm could also pollute food, potentially leading to FBD after consumption. Møller et al. [30] also found the risk of cross contamination between outside environment and home due to the high concentration of microorganisms on work clothing. Other exposures related to agricultural activities, such as manure and animal contact, may also increase the risk of foodborne zoonotic diseases. Pathogens from animals or animal products can be transferred from farms into the domestic environment and onto food, especially if recommended hygiene practices (e.g., handwashing after animal contact) are not followed.

Holding all other factors constant, Eq. (2) suggests that the optimal level of risk mitigation behavior is greater for individuals living in agricultural household *j* than individuals living in other households *i*. If education gives people the ability to better distinguish between personalized risk and general risk, as the Grossman model suggests, we expect to see a greater difference in risk mitigation efforts between agricultural and non-agricultural households for those who are educated.

#### The optimal level of health

In the previous section, we theorized that education improves the efficiency of health-related decision-making, and the optimal amount of safe food handling practices adopted is not equivalent to maximum adoption due to resource limitations. According to the Grossman health model, individuals with higher levels of education are better able to make efficient health decisions, thereby increasing their utility. Further, an individual's utility is determined by health *(H)* and the consumption of non-health goods such as entertainment equipment and household appliances *(Z)*: *u* = *u (H, Z)*. There is a resource constraint, without which individuals would have unlimited resources to improve both health and consumption of non-health goods, indicating that people would adopt safe food handling practices as much as possible. With the constraint, however, people have to decide the optimal amount of resource they spend on the two activities, health improvement and consumption of non-health goods. Therefore, we can conclude that the Grossman model predicts that individuals make tradeoffs between health and non-health goods, resulting in less than maximal health [31].

#### Optimism bias and food handling practices

An alternative source of heterogeneity in the educational effect on health behavior is optimism bias. Cogan [32] expanded on the concept of optimism bias by introducing self-serving bias, particularly in the context of food safety. This bias leads people to believe that they are less likely to experience negative events compared to others, often attributing this to factors such as perceived intellectual superiority. Evans et al. [12] indicated a positive correlation between education and optimism bias in a similar food handling study. This suggests that individuals with higher levels of education may perceive lower risks associated with inadequate or inappropriate food handling practices.

### Summary of effects

The impact of education on the adoption of safe food handling practices may be influenced by two opposing factors. First, the effect of higher level of education attainment, including improved knowledge and efficiency, leads to better and more efficient safe food handling practices. Second, the perception of risk tends to decrease with education due to optimism bias. It is important to note that, as discussed previously with the Grossman model, the term 'efficient' does not necessarily mean that individuals with higher level of education will adopt more safe food handling practices than others. This is due to the opportunity costs associated with these practices (the tradeoffs between *H* and *Z*), as the time and effort spent on them could be used for other activities considered more valuable. Rather, being “efficient” means that people with higher levels of education may have knowledge and critical thinking skills that allow them to better assess which practices are utility maximizing. As a result, the effect of education on safe food handling practices is indeterminate, meaning that education may have a positive, negative, or net zero effect on these practices. This educational effect can be illustrated through the following equations.

The probability of adopting safe food handling practice *f* is determined by *o*, level of optimism bias and *k*, knowledge, where *o* is negatively correlated with *f*, as those with larger optimism bias are less likely to adopt the safe food handling practice, while the effect of *k* can vary depending on people’s perception of risk. Those with higher level of education are more capable of accurately estimating the risk [33] and adjusting their behaviors accordingly. The relationship can be shown as:

$$\text{Given that}: f=f\left(o\left(e\right), k\left(e\right)\right),\frac{\partial k}{\partial e}>0,\frac{\partial o}{\partial e}$$ >0

3 $$\begin{array}{c}\frac{\partial f}{\partial o}<0\end{array}$$

4 $$\begin{array}{c}\frac{\partial f}{\partial k}>or=or<0\end{array}$$

5 $$\begin{array}{c}\left|\frac{\partial f}{\partial o}\right|>or=or<\left|\frac{\partial f}{\partial k}\right|\end{array}$$

6 $$\begin{array}{c}\frac{\partial f}{\partial e}=\frac{\partial f}{\partial o}\cdot \frac{\partial o}{\partial e}+\frac{\partial f}{\partial k}\cdot \frac{\partial k}{\partial e}>or =or<0\end{array}$$

The optimism bias may become smaller when an event is perceived as more likely to occur [34, 35]. For instance, in agricultural households, the perceived risk of FBD may be higher, leading to a reduction in the optimism bias among respondents in such households. Consequently, the indirect negative effect of education on safe food handling practices, which is reflected through the negative impact of optimism bias on protective behavior, may be smaller when the perceived risk is higher. The relationship is reflected in the following equation: the subscripts *l* and *h* represent low and high perceived risk, respectively:

7 $$\begin{array}{c}\frac{{\partial f}_{l}}{{\partial o}_{l}}>\frac{{\partial f}_{h}}{{\partial o}_{h}}\end{array}$$

In sum, this study contributes to the literature and builds on previous works in several ways. First, the study uses a unique dataset from Ethiopia to empirically test hypotheses based on Grossman’s model in a LMIC. Second, unlike other Ethiopian food safety evaluations, this dataset has a large sample size, focuses on safe food handling practices at home (rather than in the context of food service), and draws from urban and rural households in three diverse population centers. Consequently, the dataset is more representative of the Ethiopian population than previous studies. Finally, this study uses agriculture household (household’s primary source of income being agriculture) as a proxy of risk to estimate the heterogeneity in the association between education and safe food handling practices among people facing different levels of risk of FBD in Ethiopia, which to the best knowledge of the authors, has not been done in previous studies. By creating a better understanding of the role of education in food safety decisions at the household level, this study could inform policy decisions around food safety management and help to identify research gaps.

## Methods and materials

### Data

A cross-sectional community survey designed to explore the prevalence of diarrheal disease and potential contributing factors was conducted in Ethiopian households. According to the survey sampling method described in LaPolt et al.^1^, data were collected from October 2021 to October 2022 in Addis Ababa, Harari, and Gondar. The surveyed households were within the catchment areas of Yekatit 12 Hospital (Addis Ababa), Hiwot Fana Comprehensive Specialized Hospital (Harari), and University of Gondar Comprehensive Specialized Hospital (Gondar). Household selection was based on randomly selected GPS coordinates. To account for seasonal variations on the variables of interest, household selection was evenly distributed throughout the study period. A representative family member was enlisted to participate in the study. The data used for this study were limited to households with respondents who indicated they were also food handlers at home, since the dataset only contained information on the education of the respondents. Consequently, the number of observations was reduced to 1,679 after also excluding observations with missing data for age and income.

### Empirical model

One of the objectives of the study was to estimate the association between education and safe food handling practices. Therefore, the dependent variables were four safe food handling practices at home that could be used to improve food safety when processing animal-sourced foods. These practices include 1) washing hands before handling food; 2) washing surface before handling food; 3) boiling or filtering the water before being used to cook food; and 4) using separate utensils for vegetable and meat. The key independent variables were the different levels of education. The original education variable in the dataset was a categorical variable with 13 different levels of education. This variable was further condensed into three levels: 1) illiterate; 2) primary education; and 3) secondary education and above. Illiterate was selected to be the reference group.

Two logistic regression models were constructed for each of the dependent variables using Stata (SE 15.1). The first model assessed the relationship between education level and practice for the four safe food handling practices:

8 $$\Pr\left(y_{ij=1}\right)={G(\beta}_0+\beta_1{PrimaryEduc}_i+\beta_2{SecondaryEduc}_i+\phi X_i)$$

The second logistic regression model assessed the heterogeneous association between education and safe food handling practices among people facing different levels of risk (i.e., the interaction between education and our primary proxy for risk):

9 $$\Pr(y_{ij}=1)={F(\delta}_0+\delta_1{PrimaryEduc}_i\ast{Agriculture}_i+\delta_2{SecondaryEduc}_i\ast{Agriculture}_i+\delta_3{PrimaryEduc}_i+\delta_4{SecondaryEduc}_i+\delta_5Agriculture+\phi X_i)$$

In both models, ${y}_{ij}$ denotes whether person *i* adopts safe food handling practice *j* (= 1 if the practice is adopted). ${PrimaryEduc}_{i}$ and ${SecondaryEduc}_{i}$ are two indicator variables on the levels of education of person *i*. ${X{\prime}}_{i}$ is a vector of controls that includes agriculture household indicator (= 1 if primary source of household income is agriculture); access to improved water (defined according to the WHO [36] standard, excluding communal taps, as LaPolt et al.^2^ showed an association between communal taps and an increased risk of diarrhea); children (= 1 if number of children in the household is greater or equal to one); last month’s household income; age; marital status; and gender of the respondent (= 1 if the respondent is a female). The practices may also be influenced by unobserved factors which were captured as part of the error in the model.

### Robustness check

#### Livestock presence in household

To check the robustness of predicted association between education and safe food handling, we estimated another model using an alternative proxy for food safety risk. Since having livestock in the household has been associated with diarrhea [37, 38], it is often considered a risk factor for exposure to foodborne pathogens. Therefore, we hypothesized that the risk of contracting FBD is higher for households with livestock and used livestock presence as an alternative proxy. The model for this robustness check was formulated as follows:

10 $$\Pr\left(y_{ij}=1\right)={F(\gamma}_0+\gamma_1{PrimaryEduc}_i\ast{AnimalPresence}_i+\gamma_2{SecondaryEduc}_i\ast{AnimalPresence}_i+\gamma_3{PrimaryEduc}_i+\gamma_4{SecondaryEduc}_i+\gamma_5AnimalContact+\phi X_i)$$

where the key independent variables for this analysis are the interactions of the education indicators and a dummy variable for livestock presence (= 1 if the household has cattle, goat, sheep, and or/poultry).

Previous literature suggests that interpreting interaction terms in logit models can be challenging, as some approaches used for interpreting interaction terms in linear models do not apply to non-linear models [39]. Although odds ratios for interaction terms can be presented and understood as ratios of odds ratios (i.e., multiplicative effects) [40], they still pose challenges in providing an intuitive understanding of the results. To address this, we used Stata's post-estimation function to calculate the average marginal effect (AME) for all the regression models illustrated above. This allowed us to present the results in probabilistic terms (e.g., a one-unit increase (or a change from 0 to 1 for a dichotomous variable) in the independent variable leads to an x-percentage point increase in the outcome variable).

## Results

### Descriptive statistics

A total of 1,679 individuals were included in the analysis after excluding 154 observations with missing age (1 observation) and income (153 observations) (Table 1). The respondents were distributed nearly evenly across the three education levels. Over a third (36%) of the respondents resided in agricultural households, and 39% of the households reported having at least one type of livestock. Most respondents were married, female and had at least one child living in their household. In examining the household income distribution, we observed a concentration towards the lower end, with over 60% of the samples reporting a monthly household income of less than 4,000 Birr. Additionally, the sample predominantly comprised young to middle-aged individuals, with more than 50% falling within the 20–39 age bracket. Overall, 70% of the respondents indicated that they washed their hands before handling food; 75% washed surfaces before handling food; 21% boiled/filtered the water before being used to cook food; and 26% used separate utensils for vegetable and meat when preparing foods.

**Table 1:** Descriptive statistics of variables of interests [N (%)]

	Wash hands	Wash Surface	Boil/Filter Water	Separate Utensils	Overall (*N* = 1,679)
Level of Education					
Illiterate	457 (81)	468 (83)	145 (26)	88 (16)	565 (34)
Primary	332 (67)	372 (75)	98 (20)	115 (23)	493 (29)
Secondary	383 (62)	417 (67)	103 (17)	227 (37)	621 (37)
Livestock Presence					
Yes	534 (82)	552 (85)	173 (27)	101 (16)	648 (39)
No	638 (62)	705 (68)	173 (17)	329 (32)	1,031 (61)
Household Type					
Agriculture	504 (83)	517 (85)	170 (28)	71 (12)	608 (36)
Non-Agriculture	668 (62)	740 (69)	176 (16)	359 (34)	1,071(64)
Improved Water					
Yes	721 (66)	793 (73)	213 (20)	327 (30)	1,091 (65)
No	451 (77)	464 (79)	133 (23)	103 (18)	588 (35)
Children					
Yes	967(71)	1,042 (76)	299 (22)	319 (23)	1,370 (82)
No	205(66)	215 (70)	47 (15)	111 (36)	309 (18)
Marital Status					
Married	871(71)	932 (78)	262 (22)	282 (24)	1,197 (71)
Not Married	301(62)	325 (67)	84 (17)	148 (31)	482 (29)
Sex					
Female	1,132(70)	1,216 (76)	342 (21)	406 (25)	1,610 (96)
Male	40(58)	41 (59)	4 (6)	24 (34)	69 (4)
Household Income					
< 2,000	526(82)	526 (82)	145 (23)	95 (15)	642 (38)
2,000–4,000	341(80)	361 (85)	87 (21)	108 (25)	424 (25)
4,000–6,000	155(57)	183 (67)	58 (21)	91 (33)	272 (16)
6,000–8,000	56(40)	83 (60)	17 (12)	55 (40)	139 (8)
8,000–10,000	35(38)	41 (45)	18 (20)	42 (46)	92 (6)
> 10,000	59(54)	63 (57)	21 (19)	39 (35)	110 (7)
Age (Years)					
15–19	59(67)	63 (72)	22 (25)	17 (19)	88 (5)
20–29	422(69)	460 (76)	130 (21)	142 (23)	608 (36)
30–39	363(75)	374 (77)	93 (19)	128 (26)	484 (29)
49–49	172(70)	181 (74)	51 (21)	61 (25)	246 (15)
> 50	156(62)	179 (71)	50 (20)	82 (32)	253 (15)
Total	1,172 (70)	1,257 (75)	346 (21)	430 (26)	1,679 (100)

Descriptive statistics (frequency) for the key variables of interest are presented, with percentages shown in parentheses. The denominators for percentage calculations for the four practices are showed in the Overall column. The denominator for the percentage calculations for the Overall column is *N* = 1,679

### Association between education and safe food handling practices

In the first model, we estimated the association between education and four food handling practices. Results of the logistic regression analysis are presented in Table 2.

**Table 2:** Association between education and food handling practices for all study participants (main effects Only)

	Wash hands	Wash Surface	Boil/Filter Water	Separate Utensils
Level of Education				
Illiterate	Reference	Reference	Reference	Reference
Primary	0.82 [0.59, 1.14]	0.95 [0.67, 1.35]	0.87 [0.62, 1.20]	1.02 [0.71, 1.45]
Secondary	1.02 [0.71, 1.47]	0.91 [0.63, 1.32]	0.81 [0.55, 1.18]	1.40 [0.97, 2.01]
Household Type				
Agriculture	**1.69** [1.24, 2.32] **	**1.60** [1.15, 2.23] **	**1.74** [1.27, 2.37] **	0.44 [0.31, 0.61] **
Improved Water				
Yes	1.03 [0.80, 1.34]	1.14 [0.87, 1.49]	1.05 [0.80, 1.36]	1.19 [0.90, 1.56]
Children				
Yes	0.85 [0.62, 1.15]	1.02 [0.75, 1.39]	1.32 [0.92, 1.89]	0.69 [0.51, 0.93] *
Marital Status				
Married	**1.33** [1.03, 1.72] *	**1.41** [1.08, 1.83] **	1.18 [0.88, 1.59]	0.80 [0.61, 1.04]
Sex				
Female	1.22 [0.71, 2.08]	1.48 [0.87, 2.53]	3.56 [1.27, 9.97] *	1.03 [0.60, 1.78]
Household Income				
< 2,000	Reference	Reference	Reference	Reference
2000–4000	0.91 [0.66, 1.27]	1.29 [0.91, 1.83]	0.97 [0.71, 1.33]	1.80 [1.29, 2.50] **
4000–6000	0.32 [0.22, 0.45]	0.51 [0.35, 0.72] **	1.21 [0.83, 1.76]	2.10 [1.45, 3.03] **
6000–8000	0.17 [0.11, 0.26]	0.40 [0.26, 0.61] **	0.72 [0.41, 1.28]	2.26 [1.45, 3.52] **
8000–10000	0.17 [0.10, 0.28] **	0.22 [0.14, 0.37] **	1.24 [0.69, 2.23]	2.87 [1.73, 4.74] **
> 10,000	0.31 [0.19, 0.49] **	0.39 [0.24, 0.62] **	1.35 [0.78, 2.37]	1.77 [1.08, 2.89] *
Age [Years]				
15–19	Reference	Reference	Reference	Reference
20–29	1.12 [0.65, 1.91]	1.19 [0.69, 2.07]	0.75 [0.43, 1.30]	1.35 [0.74, 2.48]
30–39	1.33 [0.76, 2.33]	1.12 [0.63, 1.97]	0.61 [0.35, 1.09]	1.86 [1.00, 3.46] *
49–49	0.96 [0.53, 1.74]	0.92 [0.51, 1.69]	0.68 [0.37, 1.24]	1.66 [0.86, 3.19]
> 50	0.73 [0.41, 1.31]	0.93 [0.51, 1.69]	0.77 [0.42, 1.41]	2.07 [1.09, 3.95] *
Baseline Odds	2.74 [1.20, 6.26] *	1.74 [0.76, 4.00]	0.06 [0.02, 0.21] **	0.20 [0.08, 0.47] **

Odds ratios are presented, with 95% confidence interval shown in parentheses

^**^ p-value < *0.01*

^*^p-value < *0.05*

Generally, the odds ratios for education variables, including the primary education and secondary education indicators, were not statistically significant at the 0.05 level. Although this appears to undermine the argument that education is associated with safe food handling practices, this result is likely an artifact of an overly simplistic model. Notably, we observed that the odds ratios for the agriculture indicator were significant and greater than one for washing hands (AOR: 1.69, CI: [1.24, 2.32]), washing surfaces (AOR: 1.60, CI: 1.15, 2.23), and boiling or filtering water (AOR: 1.74, CI: [1.27, 2.37]). We also found that married individuals were more likely to wash their hands (AOR:1.33, CI: [1.03, 1.72]) and wash surfaces before preparing animal-sourced foods (AOR: 1.41, CI: [1.08, 1.83]). Additionally, respondents living in households with higher monthly incomes had a lower likelihood of adopting these two practices but more likely to use separate utensils compared to those with household monthly incomes less than 2,000 Birr. However, income and marital status were not associated with boiling or filtering water.

### Heterogeneous association among households facing different FBD risks

#### Illustration of results

In this section, we examined how exposure to agriculture modifies the role of education in the adoption of safe food handling practices. Our primary analysis focused on agriculture households, though we also assessed livestock presence as an alternative proxy for agriculture exposure.

The odds ratios for the interactions between agriculture and education were statistically significant and greater than one in the models for washing hands before handling animal-sourced food (AgPrimaryEdu: AOR: 3.11, CI: [1.54, 6.29]); AgSecondaryEdu: (AOR: 8.67, CI: [2.69, 28.00]) and washing surface before preparing animal-sourced food (AgPrimaryEdu: AOR: 2.15, CI: [1.04, 4.45]); AgSecondaryEdu: AOR: 8.06, CI: ([2.21, 29.43]) as shown in Table 3. These findings suggested that the association between education and these two practices was heterogeneous among individuals with and without exposure to agriculture. Additionally, we found that education was negatively associated with the adoption of these practices for non-agricultural households, as indicated by odds ratios smaller than one. Similar results were found for boiling or filtering water, where the odds ratio for the interaction between secondary education and agriculture was greater than one, while the odds ratio for the main effect of secondary education was less than one.

**Table 3:** The heterogeneous association between education and safe food handling (agricultural household as a proxy)

	Wash hands	Wash Surface	Boil/Filter Water	Separate Utensils
Agriculture* Primary Education	**3.11** [1.54, 6.29] **	**2.15** [1.04, 4.45] **	1.64 [0.86, 3.10]	1.08 [0.51, 2.31]
Agriculture* Secondary Education	**8.67** [2.69, 28.00] **	**8.06** [2.21, 29.43] **	2.35 [1.09, 5.04] *	2.23 [1.00, 5.00] *
Primary Education	0.45 [0.28, 0.73] **	0.62 [0.38, 1.01]	0.63 [0.39, 1.02]	0.93 [0.60, 1.43]
Secondary Education	0.57 [0.36, 0.92] *	0.58 [0.36, 0.93] *	0.57 [0.35, 0.91] *	1.19 [0.78, 1.82]
Household Type				
Agriculture	0.80 [0.49, 1.30]	0.90 [0.55, 1.49]	1.22 [0.78, 1.90]	0.36 [0.22, 0.58] **
Improved Water				
Yes	1.04 [0.80, 1.35]	1.14 [0.87, 1.50]	1.05 [0.80, 1.37]	1.18 [0.90, 1.56]
Children				
Yes	0.84 [0.62, 1.15]	1.02 [0.75, 1.39]	1.32 [0.91, 1.89]	0.69 [0.51, 0.93] *
Marital Status				
Married	1.37 [1.06, 1.78] *	1.45 [1.11, 1.89] **	1.20 [0.89, 1.62]	0.81 [0.62, 1.05]
Sex				
Female	1.28 [0.74, 2.21]	1.56 [0.91, 2.67]	3.67 [1.31, 10.32] *	1.05 [0.61, 1.82]
Household Income				
< 2,000	Reference	Reference	Reference	Reference
2,000–4,000	0.90 [0.65, 1.25]	1.28 [0.90, 1.82]	0.96 [0.70, 1.32]	1.80 [1.29, 2.50] **
4,000–6,000	0.32 [0.22, 0.45] **	0.51 [0.36, 0.73] **	1.23 [0.85, 1.80]	2.12 [1.46, 3.05] **
6,000–8,000	0.18 [0.11, 0.27] **	0.41 [0.27, 0.64] **	0.75 [0.42, 1.33]	2.30 [1.47, 3.58] **
8,000–10,000	0.18 [0.11, 0.29] **	0.23 [0.14, 0.39] **	1.32 [0.73, 2.39]	2.95 [1.78, 4.88] **
> 10,000	0.33 [0.20, 0.52] **	0.41 [0.26, 0.66] **	1.43 [0.82, 2.51]	1.83 [1.12, 2.99] *
Age [Years]				
15–19	Reference	Reference	Reference	Reference
20–29	1.20 [0.69, 2.07]	1.25 [0.72, 2.18]	0.77 [0.45, 1.34]	1.36 [0.74, 2.48]
30–39	1.47 [0.83, 2.60]	1.19 [0.67, 2.13]	0.64 [0.36, 1.14]	1.89 [1.02, 3.51] *
49–49	1.08 [0.59, 1.97]	1.00 [0.54, 1.85]	0.71 [0.39, 1.32]	1.69 [0.88, 3.26]
> 50	0.78 [0.43, 1.41]	0.96 [0.53, 1.77]	0.79 [0.43, 1.45]	2.08 [1.09, 3.95] *
Baseline Odds	3.90 [1.63, 9.31] **	2.24 [0.94, 5.35]	0.08 [0.02, 0.26] **	0.21 [0.09, 0.51] **

Odds ratios are presented, with 95% confidence interval shown in parentheses

^**^ p-value < *0.01*

^*^p-value < *0.05*

AME results, which provide a more intuitive interpretation of the findings are shown in Appendix I. For washing hands, secondary education attainment was associated with a 20(CI: [10,28]) percentage points increase in the probability of adopting this practice compared to the reference group (illiterate) in agricultural households. However, for non-agricultural households, secondary education was associated with a 10(CI: [−18, −2]) percentage points decrease in probability. For primary education, a negative association was found (AME: −0.15, CI: [−0.23, −0.06]) within non-agricultural households, while a positive association (AME: 0.06, CI: [−0.03,0.14]) was observed for agricultural households, though it was not significant at the 0.05 level. For washing surfaces, secondary education was associated with a 16(CI: [8,24]) percentage points increase in the probability of adopting this practice in agricultural households and a 10(CI: [−17, −2]) percentage points decrease in non-agricultural households. Similar patterns were found for primary education, with a predicted increase in adoption (AME: 0.04, CI: [−0.04,0.12]) in agricultural households, though this was not significant at the 0.05 level, and a decrease in non-agricultural households (AME: −0.08, CI: [−0.16, −0.001]).

For the other two practices, the association between education and the probability of adopting safe food handling practices was less pronounced. Secondary education was associated with a lower probability of boiling or filtering water (AME: −0.08, CI: [−0.16, −0.01]) in non-agricultural households and a higher probability of using separate utensils (AME: 0.15, CI: [0.02,0.28]) in agricultural households. The association between primary education and the probability of adopting food handling practices was not significant.

We also examined the contrast in the probability of adopting safe food handling practices between individuals with secondary education and above and those with primary education. We found that the difference in the probability of adopting these practices was statistically significant for washing hands, washing surfaces, and using separate utensils among agricultural households. Individuals with secondary education and above were also more likely to adopt these practices compared to those with primary education within agricultural households.

#### Explanation of results

Results were consistent with the hypothesis that individuals in agricultural households were more likely to adopt practices that could improve health and overall utility. However, the main effect of education for non-agricultural households appeared to be negative for these practices (for washing surfaces, the negative association was only significant for secondary education). Though surprising, this is consistent with our prediction that education does not necessarily increase the likelihood of adopting safe food handling practices due to the trade-offs people must make to maximize utility. Education helps people understand and estimate the risks they may face so that they adopt an optimal level of risk-mitigating practices (e.g., adopting a lower level of handwashing when the risk was low).

It is also possible that in environments where health investments yield significant returns (e.g., washing hands significantly improves health when food safety risk is high), individuals with higher levels of education are more likely to adopt safe food handling practices, such as washing hands and washing surfaces, since they better understand food safety risks and the benefits of allocating resources to protective health measures when health investment is efficient.

However, behavioral biases may have interfered with this process by encouraging irrationality. Previous studies showing a positive correlation between education and optimism bias suggested that optimism bias could be negatively associated with the likelihood of adopting safe food handling practices. The positive association between education and safe food handling practices in agricultural households indicated that the optimism bias was smaller when the risk of contracting disease was high. The greater than one odds ratios for the interaction terms indicated that the negative association between education and safe food handling practices, which may have been driven by optimism bias, was mitigated in agricultural households. These findings supported the previously established relationship that the indirect negative association between education and safe food handling practices, reflected through optimism bias, was smaller for those with higher perceived risks.

Conversely, in low-risk environments where health investment was considered inefficient, individuals with higher levels of education may have preferred to allocate resources to other non-health goods, making them less likely to adopt certain protective practices compared to those with lower levels of education. The optimism bias amplified this negative association, resulting in an overall negative association between education and safe food handling for non-agricultural households.

### Robustness check

#### Livestock presence in household

Results using livestock presence in the household as a proxy for food safety risk (Table 4) were similar to those when agricultural household indicator was used as a proxy for food safety risk. Livestock presence was not completely exogenous and there was a moderate correlation of 0.69 between the agricultural household indicator and the livestock presence. Individuals in agricultural households were also more likely to have livestock.

**Table 4:** The heterogeneous association between education and safe food handling (livestock presence as a proxy)

	Wash hands	Wash Surface	Boil/Filter Water	Separate Utensils
Livestock * Primary Education	**3.72** [1.88, 7.36] **	**3.10** [1.51, 6.38] **	1.50 [0.81, 2.80]	1.02 [0.50, 2.08]
Livestock * Seconadary Education	**10.49** [4.50, 24.46] **	**10.78** [4.20, 27.65] **	1.63 [0.84, 3.16]	2.47 [1.29, 4.75] **
Primary Education	**0.40** [0.25, 0.63] **	**0.52** [0.32, 0.83] **	0.63 [0.40, 1.00]	1.09 [0.71, 1.68]
Secondary Education	**0.45** [0.28, 0.71] **	**0.45** [0.28, 0.71] **	0.56 [0.35, 0.88] *	1.36 [0.89, 2.07]
Livestock Presence				
Yes	0.66 [0.41, 1.07]	0.74 [0.45, 1.21]	1.63 [0.84, 3.16]	0.49 [0.30, 0.79] **
Improved Water				
Yes	1.06 [0.81, 1.39]	1.18 [0.89, 1.55]	1.04 [0.80, 1.36]	1.22 [0.93, 1.61]
Children				
Yes	0.81 [0.59, 1.10]	0.97 [0.71, 1.33]	1.30 [0.90, 1.87]	0.68 [0.50, 0.92] **
Marital Status				
Married	1.36 [1.05, 1.77] *	1.43 [1.10, 1.87] *	1.21 [0.90, 1.62]	0.78 [0.60, 1.02]
Sex				
Female	1.43 [0.82, 2.52]	1.76 [1.01, 3.07] *	3.77 [1.34, 10.61] *	1.06 [0.61, 1.84]
Household Income				
< 2,000	Reference	Reference	Reference	Reference
2,000–4,000	0.86 [0.62, 1.19]	1.22 [0.86, 1.74]	0.94 [0.69, 1.29]	1.81 [1.30, 2.51] **
4,000–6,000	0.29 [0.21, 0.42] **	0.48 [0.33, 0.69] **	1.17 [0.80, 1.70]	2.16 [1.50, 3.12] **
6,000–8,000	0.17 [0.11, 0.26] **	0.41 [0.26, 0.63] **	0.72 [0.40, 1.27]	2.42 [1.56, 3.77] **
8,000–10,000	0.17 [0.10, 0.28] **	0.23 [0.14, 0.38] **	1.22 [0.68, 2.19]	3.19 [1.92, 5.28] **
> 10,000	0.32 [0.20, 0.51] **	0.40 [0.25, 0.65] **	1.33 [0.76, 2.32]	1.97 [1.21, 3.21] **
Age [Years]				
15–19	Reference	Reference	Reference	Reference
20–29	1.24 [0.72, 2.14]	1.33 [0.76, 2.33]	0.76 [0.44, 1.31]	1.42 [0.78, 2.60]
30–39	1.53 [0.86, 2.70]	1.27 [0.71, 2.27]	0.61 [0.34, 1.08]	2.03 [1.10, 3.78] *
49–49	1.09 [0.60, 2.00]	1.04 [0.56, 1.92]	0.68 [0.37, 1.25]	1.79 [0.93, 3.45]
> 50	0.77 [0.43, 1.40]	0.99 [0.54, 1.82]	0.74 [0.40, 1.35]	2.28 [1.20, 4.33] **
Baseline Odds	4.08 [1.70, 9.74] **	2.28 [0.95, 5.47]	0.08 [0.02, 0.27] **	0.16 [0.07, 0.39] **

Odds ratios are presented, with 95% confidence interval shown in parentheses

^**^ p-value < *0.01*

^*^p-value < *0.05*

Significant odds ratios were found for the interaction terms in the models for three practices: washing hands, washing surfaces, and using separate utensils. These findings suggest that the heterogeneous association between education and safe food handling is consistent when using different proxies to differentiate the perceived risk of contracting diseases among groups.

The post-estimation AME analysis (Appendix I) supported the conclusions mentioned above. We found a significant positive association between education and safe food handling practices, specifically washing hands and washing surface, for households with livestock. In contrast, for those without livestock, education was negatively associated with the probability of adopting these practices. Additionally, the associations were less pronounced for boiling or filtering water and using separate utensils, which was consistent with our earlier findings when using agricultural indicator as a proxy.

## Discussion

Our results indicate that education is heterogeneously associated with the adoption of two safe food handling practices: washing hands and washing surface. Education seems to be negatively associated with these practices among individuals in non-agricultural households. However, this negative association diminishes and becomes more positive for those in agricultural households. The results align with the Grossman model’s prediction that individuals make tradeoffs between health and non-health goods, resulting in less than maximal health. With limited resources, individuals must choose the optimal level of health to maximize utility rather than adopting the health practices at a maximum level. Individuals with higher levels of education may be more capable of accurately estimating risk and choosing the optimal level of safe food handling practices accordingly.

Optimism bias, which has previously been shown to be increased with education, may interfere with this process by either amplifying the negative association between education and safe food handling when health investment is inefficient (e.g., when the health risks are lower) or counteracting the positive association when health investment yields significant returns (e.g., in high-risk contexts). These results are, to some extent, consistent across different proxies, as demonstrated in the robustness check.

Additionally, the odds ratios for the interaction between education and agriculture and boiling or filtering water and using separate utensils models were not significant. A possible explanation is that these practices differ in nature from washing hands and washing surface. One of the reasons why people wash their hands and surfaces is to prevent cross-contamination between the outdoor environment and food, thereby reducing the risk of contracting diseases from bringing in outside contaminants (e.g., contaminated substances from farmland) into their home [41]. We hypothesized that agricultural households are at higher risk of FBD due to the likelihood of bringing in contaminated objects because of their occupation [29, 30].

However, boiling and filtering water and using separate utensils are not aimed at preventing cross-contamination between the outside environment and food at home. The primary purpose of boiling and filtering water is to eliminate pathogens and harmful substances, while using separate utensils aims to prevent cross-contamination between uncooked and ready-to-eat foods (e.g., leafy greens). The ready-to-eat foods have also been identified as a source of FBD, leading to significant economic losses [42]. It is possible that the difference in cross-contamination risk between uncooked and ready-to-eat foods for agricultural versus non-agricultural households was not perceived to be as large as the difference in the risk of bringing outside contaminants into the household. It is also possible that the adoption of these two practices was influenced by the access to appliances (e.g., access to filter was limited or only one set of utensils was available). As a result, the heterogeneous associations were not as pronounced for these two practices. Further studies need to be conducted to analyze the underlying reasons why the relationship between education and food handling behaviors was different for these two practices.

### Policy implications

The primary finding of this study is that education is differentially associated with self-reported safe food handling practices. Specifically, education has a positive association with safe food handling practices within agricultural households. One implication of this finding is that education is particularly effective in increasing the adoption of safe food handling practices when handling animal-sourced foods in risky environments or when people have a higher perception of the risk of being infected with foodborne diseases. Where resources for training are scarce, it may make sense to use educational campaigns to focus on those most likely to benefit from the knowledge conveyed. In this case, agricultural households and households with livestock have been shown to be more responsive to risk, as supported by the positive and non-trivial average marginal effects shown in the results, and thus, could benefit from targeted education.

Improving risk communication may help increase the adoption of safe food handling practices. The knowledge, attitude, and practice (KAP) theory [43] suggests that behavioral change occurs in three steps: the formation of knowledge, a change in attitude, and finally, a change in behavior. These insights imply that interventions aimed at enhancing people's attitudes (e.g., risk perception) could amplify the educational impact and increase the adoption of proper food handling practices, if that is the ultimate goal. When forming policies to increase people’s risk perception, it is important to consider that people may not have accurate information about the risk of FBD. While people are probably aware of the prevalence of FBD, they may not fully grasp the severity of these diseases. For example, diarrhea, a common symptom of FBD, is often self-limiting [44] and may not be viewed as a serious threat even though it can be fatal, especially for children under five years old [1]. Therefore, applying the "fear appeals" approach could be helpful as a method to increase people’s perception of risk (e.g., severity) and change their behaviors [45]. Some studies conducted in LMICs have shown that fear appeals increase the adoption of protective behaviors [46, 47].

Even if people are fully informed about the general risk of FBD, we should consider other factors, such as optimism bias, that may influence their behavior and perception of group-specific risk. This bias often occurs when individuals do not see themselves as part of a stereotypical group that is vulnerable to negative events [34]. In the context of this study, this could involve individuals who do not perceive themselves as likely to contract FBD. Optimism bias leads people to compare their situations with the average person rather than with specific individuals, which may result in the creation of the so-called “better than average” effect [48] and could lead to an underestimation of the actual risk of contracting FBD. A previous study on food handling practices suggested that optimism bias was positively associated with the level of education [12], indicating that individuals with higher levels of education may be more likely to underestimate risks and, as a result, less likely to adopt safe food handling practices. In this case, we should encourage more individualized comparisons to reduce over-optimism. Specifically, connecting individuals with similar socioeconomic backgrounds who differ in their adoption of protective practices could help increase the adoption of proper food handling practices. A more specific example could be that if we believe that high-income, well-educated individuals in non-agricultural households do not wash their hands frequently enough, a government advertisement could feature someone with an advantaged socio-economic background who frequently washes their hands to reduce food safety risks, serving as a positive role model. We can also provide information to households facing different levels of FBD risk about group-specific risk, for example, highlighting different risks for agricultural versus non-agricultural residents, so that people will have a more accurate reference point when making comparisons. This approach could help reduce optimism bias, improve the accuracy of individual risk assessment, and thus increase the adoption of safe food handling practices.

### Limitations

These results are suggestive, meaning that the study design does not allow us to directly test the causal relationships proposed in the theoretical framework. For example, although we observe a negative association between education and the probability of adopting certain safe food handling practices for non-agricultural households, we cannot determine causality since this is an observational study and the independent as well as dependent variables were measured simultaneously.

Additionally, the use of proxies was based on the hypothesis that rural households with intensive participation in agricultural activities and a higher likelihood of contact with livestock face an increased risk of exposure to foodborne pathogens compared to those without intensive agricultural participation and contact with livestock. Although this has been suggested in previous literature, it should be further tested within the context of the current study. Also, other factors that differently affect the behaviors of agricultural and non-agricultural households may undermine the strength of the proxies which were primarily used as indicators of risk. Future studies focusing more on behavioral bias and risk (e.g., including questions related to risk preference) may contribute to a more comprehensive understanding of the mechanisms underlying the observed relationships. We also noted that safe practices require an enabling environment, which is sometimes lacking in LMICs (e.g., limited access to necessary infrastructure). Although we controlled for some of these factors in the model, unobserved factors may still affect the associations analyzed in the study.

Moreover, for each of the above models that tested the heterogeneous association, we also examined specifications that were controlled for region. For the sample we are using, region is a problematic control variable because regions are highly correlated with key risk factor variables (agriculture and livestock presence). For example, only one of the households in our working sample from the Addis Ababa region is associated with agricultural employment. Given this high correlation between region and the key risk factors, we were not surprised to find that the inclusion of region eliminated the significance of the odds ratios for the interaction of risk and education for agricultural households, though some significant odds ratios for livestock presence persisted. To completely disentangle the influence of region on risk factors, we would need a sample that has a more even balance of risk factors across regions.

Finally, the study was conducted exclusively in three regions in Ethiopia, which may limit its generalizability to other parts of the world. Nevertheless, because our results are consistent with theory, future research in other regions may yield the same result.

## Conclusions

The study found that education was associated with a higher adoption of safe food handling practices among individuals living in agricultural or livestock-owning households. Given the greater risk faced by these households, our results suggest that risk perception may play a significant role in influencing the relationship between education and safe food handling practices. More generally, we show how future policies aimed at promoting the adoption of safe food handling practices may benefit from considering individuals' decision-making processes and behavioral biases in relation to varying risk levels.

## Data Availability

The datasets used and analyzed during the current study are available from the corresponding author on reasonable request.

## Appendix

**Table 5:** Average marginal effect of key variables

**Level of Education**	Agriculture	Non-Agriculture	Livestock Presence	No Livestock Presence
**Wash Hands**				
Primary	0.06 [−0.03,0.14]	**−0.15 **** **[-.0.23, −0.06]**	0.06 [−0.02,0.15]	**−0.17 **** **[−0.25, −0.09]**
Secondary and above	**0.20 **** **[0.10.0.28]**	**−0.10 *** **[−0.18, −0.02]**	**0.20 **** **[0.12,0.27]**	**−0.14 **** **[−0.22, −0.07]**
**Wash Surface**				
Primary	0.04 [−0.04,0.12]	**−0.08 *** **[−0.16, −0.001]**	0.07 [−0.01,0.15]	**−0.11 **** **[−0.18, −0.04]**
Secondary and above	**0.16 **** **[0.08,0.24]**	**−0.10 *** **[−0.17, −0.02]**	**0.17 **** **[0.10, 0.23]**	**−0.14 **** **[−0.21, −0.06]**
**Boil/Filter Water**				
Primary	0.00 [−0.08,0.09]	−0.07 [-.015,0.01]	−0.01 [−0.09,0.07]	−0.07 [−0.15,0.00]
Secondary and above	0.06 [−0.07,0.19]	**−0.08 *** **[−0.16, −0.01]**	−0.02 [−0.11,0.08]	**−0.09 *** **[−0.16, −0.02]**
**Use Separate Utensils**				
Primary	0.00 [−0.07,0.07]	−0.01 [−0.10,0.07]	0.01 [−0.06,0.09]	0.02 [−0.06,0.09]
Secondary and above	**0.15 *** **[0.02,0.28]**	0.04 [−0.05,0.12]	**0.21 **** **[0.11,0.30]**	0.06 [−0.03,0.14]

^****^*P value* < *0.01*

^***^*P value* < *0.05*

## Abbreviations

FBD: Foodborne disease
LMICs: Low- and middle-income countries
CDC: The U.S. Centers for Disease Control and Prevention
WHO: The World Health Organization

## Footnotes

1 LaPolt D, Mego L, Alonso S, Azmeraye BM, Teshale AM, Ayana GM, Angaw DA, Degefaw D, Garsow AV, Beczkiewicz A, Yimer G, Krakowski M, Scharff R, Seyoum ET, Kowalcyk B. Prevalence of diarrheal illness and characterization of healthcare-seeking behavior in Ethiopia: A community-based study. [Unpublished Manuscript]. Forthcoming 2024. LaPolt D, Gazu L, Alonso S, et al. TARTARE Household Diarrheal Diseases Data from Three Communities, Ethiopia, 2021–2022 [Dataset]. Forthcoming 2024. The study as well as the dataset are currently under journal review.

2 LaPolt D, Smith S, Gazu L, Alonso S, Teshale AM, Azmeraye BM, Mamo G, Abebaw D, Degefaw D, Garsow AV, Beczkiewicz A, Yimer G, Krakowski M, Scharff R, Seyoum E, Kowalcyk B.a. Environmental risk factors associated with community diarrheal disease in Ethiopia. [Unpublished Manuscript]. 2024.
